# Plant chloroplasts enable high-yield and cost-effective production of the engineered bacterial enzyme CueO-MacHis as a technical protein via transient expression in *Nicotiana benthamiana*

**DOI:** 10.3389/fpls.2026.1907380

**Published:** 2026-08-19

**Authors:** Henrik Nausch, Catherine Rose Bernau, Dirk Scheffler, Jörg Ingmar Schuphan

**Affiliations:** Department Model-based Product and Bioprocess Engineering, Fraunhofer Institute for Molecular Biology and Applied Ecology (IME), Aachen, Germany

**Keywords:** bacterial protein, *Nicotiana benthamiana*, plant molecular farming, technical enzymes, transient expression

## Abstract

**Introduction:**

Laccases are versatile oxidoreductases found in bacteria, fungi, and plants., which have a wide range of industrial applications, including the removal of pharmaceutical micropollutants from wastewater. Bacterial laccases are particularly attractive because of their high catalytic activity and stability over broad temperature and pH ranges. One example is the copper efflux oxidase (CueO) from *Escherichia coli*, which has been fused to the adhesion-promoting peptide Macaque Histatin (MacHis) because immobilized enzymes have proven especially effective for solid-phase wastewater treatment. However, the practical application of this enzyme is constrained by the relatively low production yields of 25–50 mg/L when expressed in recombinant *E. coli* or *Pichia pastoris*.

**Methods and results:**

Here, we used transient expression in Nicotiana benthamiana to demonstrate that the engineered CueO-MacHis can be produced at substantially higher levels, reaching 250–320 (286 ± 35) mg/kg biomass in the prokaryotic-like environment of chloroplasts in 6-week-old plants, which are typically used for production purposes. Subsequent systematic optimization of the transient expression process based on statistical experimental designs increased the accumulation of recombinant CueO-MacHis approximately threefold to 846 ± 57 mg/kg biomass. Furthermore, we established a simplified and cost-effective purification strategy that leverages the enzyme’s temperature and pH stability to clarify extracts before anion exchange chromatography, achieving 76% recovery and 92% purity. Moreover, the plant-derived CueO-MacHis had a specific activity of 7.22 ± 1.55 U/mg, which is in the same range as that reported for other CueO enzymes.

**Discussion:**

Our approach paves the way for the large-scale production of CueO-MacHis as a technical enzyme for industrial applications.

## Introduction

Micropollutants in municipal wastewater, especially pharmaceuticals (e.g., antibiotics, analgesics, antidepressants, beta-blockers, and hormones), are a major concern because a substantial fraction is persistent and can affect ecosystems as well as the health, development, and reproduction of humans (Pinto et al., 2022; Wilkinson et al., 2022; Gupta et al., 2024; Paíga et al., 2025; Wołowicz and Munir, 2025). Conventional wastewater treatment plants (WWTPs) based on activated sludge only partially remove micropollutants, allowing relevant amounts to accumulate in the environment with increasingly negative impacts (Park et al., 2020; Khasawneh and Palaniandy, 2021; McLachlan et al., 2022; Chaber-Jarlachowicz et al., 2025; Ianes et al., 2025). Authorities worldwide therefore require a quaternary treatment step, such as adsorption with activated carbon, membrane-based ultrafiltration and nanofiltration, or oxidation by ozonation or UV/H_2_O_2_ (Belete et al., 2023; Jurík et al., 2024; Nagy-Mezei et al., 2024). However, these treatments are associated with high capital and operating costs and/or generate extremely hazardous waste, and therefore are not considered sustainable (Tarpani and Azapagic, 2018; Pistocchi et al., 2022; Tarpani and Azapagic, 2023). For example, activated carbon and ultrafiltration membranes are expensive and generate highly contaminated waste streams, whereas ozonation is highly energy-intensive. Accordingly, alternative methods focusing on enzymatic approaches are needed (Feng et al., 2021; Ahmad et al., 2024; Feng et al., 2025).

Against this background, bacterial laccases are used for the detoxification of pharmaceutical residues in wastewater by oxidation because of their broad substrate specificity, rapid and highly efficient oxidative activity under the conditions present in wastewater, high stability, and tolerance to harsh conditions such as high concentrations of halide ions (Arregui et al., 2019; Paraschiv et al., 2022; Chen et al., 2023; Gałązka et al., 2023; Rahman et al., 2024; Saxena et al., 2024; Thathola et al., 2024). One of these laccases is the *Escherichia coli* copper efflux oxidase (CueO), a multicopper oxidase (MCO), which was recently engineered for industrial applications (Novoa Henríquez, 2019; Zhang et al., 2020; Nenninger et al., 2025). Because immobilized enzymes are particularly effective for wastewater treatment (Hedar et al., 2023; Maghraby et al., 2023; Rodríguez-Couto, 2024; Ud Din Sheikh et al., 2025), CueO has been fused to the Macaque Histatin (MacHis) adhesion-promoting peptide (Nenninger et al., 2025).

Such technical and industrial enzymes are typically produced in microbial hosts by fermentation in bioreactors (Singh et al., 2016; Fasim et al., 2021). Although well established and cost-effective, bioreactor-based production is resource- and energy-intensive because of the bioreactor infrastructure, the substantial amounts of carbon, nitrogen, and water needed for the cultivation medium, and the electrical power required for optimal fermentation conditions (i.e., temperature, pH, aeration, and agitation) (Becker et al., 2021; Basaran, 2024).

The production of recombinant technical enzymes in plants offers a less expensive and more sustainable alternative known as plant molecular farming (PMF). Despite the potential economic advantages of plant-based production and the large market volume of technical proteins and industrial enzymes, they have received less attention, and PMF research and commercial activities have been dominated by high-value biopharmaceutical proteins thus far. This shift from high-value biopharmaceuticals to high-volume industrial proteins requires production platforms that combine high productivity with economically viable upstream production (USP) and downstream processing (DSP). Demonstrating the viability of plants as a production platform for technical enzymes therefore represents an important step toward broadening the technological and industrial application scope of PMF.

Transgenic (stably transformed) plants can be grown in either greenhouses or open fields with nearly unlimited scalability using standard agricultural practices while consuming far fewer resources than bioreactor-based fermentation (van Beilen et al., 2007; Tusé et al., 2014; Nausch et al., 2016; Zhu et al., 2017; Schmidt et al., 2019; Kelada et al., 2021; Huckauf et al., 2022; Basaran, 2024). In the case of open-field cultivation, depending on the intended application and the applicable regulatory requirements, appropriate biosafety measures are applied to minimize the potential for gene flow to compatible relatives and to enable large-scale crop production. Commercial cultivation of genetically modified crops has been successfully implemented for decades in North and South America under established regulatory frameworks and crop management strategies, demonstrating that this potential risk can be effectively managed. For example, transgenic plants were grown on 216 million hectares globally in 2025, representing 65%–70% of cropland in Brazil and Argentina and 40%–45% in the United States, which are the leading countries for the cultivation of genetically modified crops (mainly soybean, maize, and cotton) (Statista, 2026). Consequently, open-field cultivation remains the envisioned production scenario for large-volume, price-sensitive technical enzymes, where agricultural-scale production constitutes one of the principal economic advantages of PMF.

Transgenic crop plants such as rice, maize, barley, potato, and tobacco can be used for the production of technical and industrial enzymes (van Beilen et al., 2007; Tusé et al., 2014; Nausch et al., 2016; Zhu et al., 2017; Schmidt et al., 2019; Kelada et al., 2021; Huckauf et al., 2022; Basaran, 2024). Among these species, tobacco, with the two species *Nicotiana tabacum* and *Nicotiana benthamiana*, is preferred because (i) it is not a food crop, eliminating the risk of unintended mixing; (ii) although it was grown on 3.3 million hectares of land worldwide in 2024 (Statista, 2025), the decline of smoking frees tobacco for other purposes but does not increase competition with food or feed crops; (iii) it has a high biomass yield of 140–165 tons fresh mass (FM) or 16–32 tons dry mass (DM) per hectare per year; (iv) the leaves can produce up to 6 g/kg FM of recombinant protein; and (v) the residual biomass is suitable for the production of green hydrogen or biochar. In addition to the time-consuming generation of transgenic plants, transient transformation (also known as transient expression) methods can be used to establish and optimize the production process more rapidly and to produce recombinant proteins in a scalable manner in greenhouse facilities.

One of the main bottlenecks of PMF is the extraction of recombinant proteins from plant biomass because target proteins are typically not secreted but instead are produced intracellularly and must be released by cellular disruption (Wilken and Nikolov, 2012; Buyel et al., 2015; Dixon et al., 2018). The released product must then be separated from the abundant solid cell debris and soluble host cell proteins (HCPs), secondary metabolites, and other impurities using filtration and chromatography methods.

Here, we employed transient expression in *N. benthamiana* to demonstrate that the bacterial laccase CueO-MacHis can be produced in plants, and we optimized both USP and DSP by applying statistical design-of-experiments (DoE) methodology to obtain an economically viable large-scale production process. To ensure that the process is suitable for future industrial implementation, the key experiments were carried out under representative preparative-scale conditions. The study therefore emphasizes process development and engineering feasibility rather than the statistical hypothesis testing of laboratory-scale experiments, providing a basis for subsequent process scale-up and future techno-economic analysis (TEA) and life cycle assessment (LCA).

## Materials and methods

### Construction of the plant transformation vector

**Figure 1:**
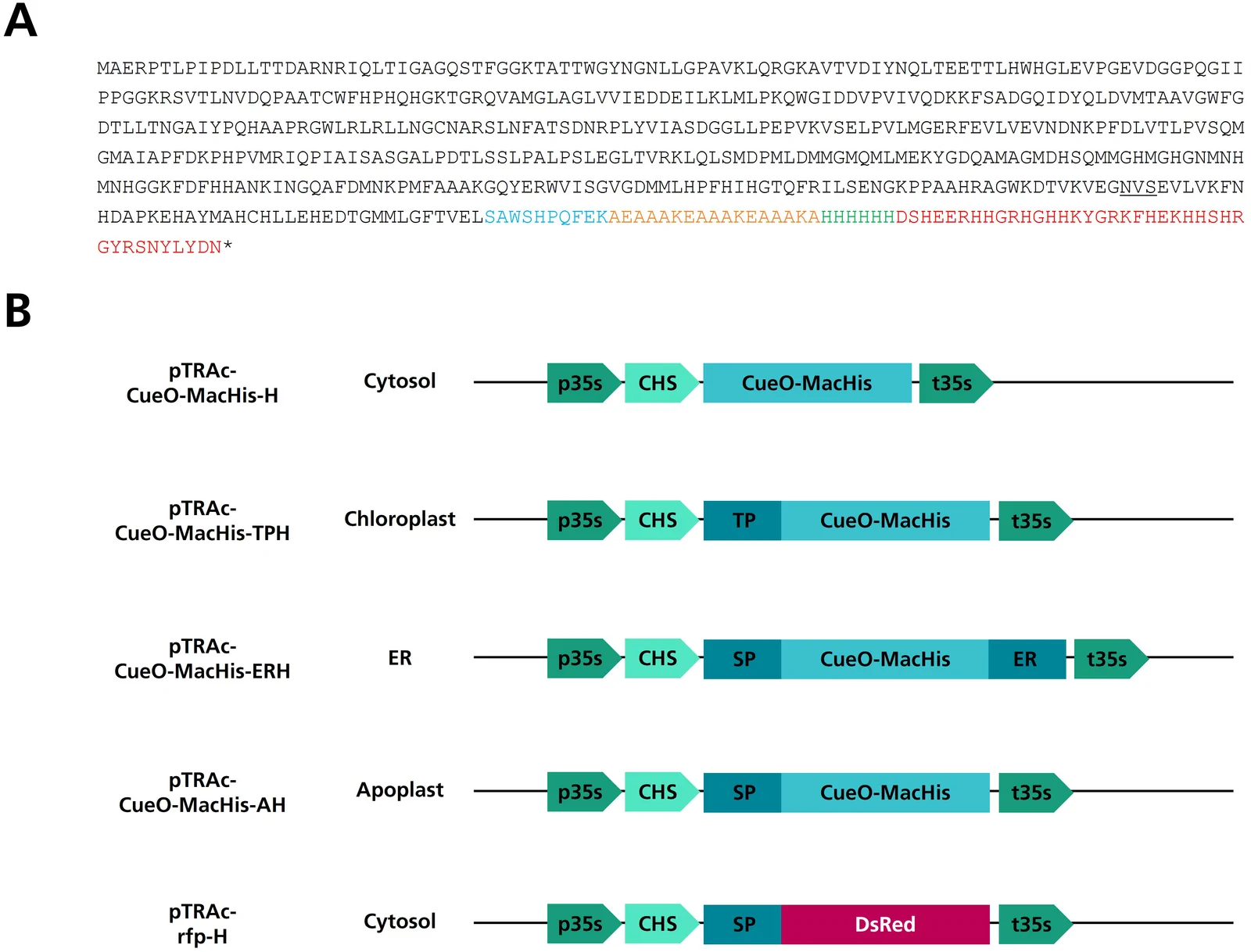
CueO-MacHis and DsRed expression constructs for transient transformation of *N. benthamiana*. **(A)** Amino acid sequence of modified CueO-MacHis: *black*, coding region of CueO; *light blue*, coding region of the Strep-tag-II; *orange*, coding region of the EA-linker; *green*, His-tag; *red*, MacHis adhesion-promoting peptide; *underlined*, potential *N*-glycosylation site. **(B)** Schematic representation of the coding region and coding region elements that lead to the expression in the cytosol, chloroplast, ER, and apoplast.

The CueO-MacHis sequence (Novoa Henríquez, 2019; Zhang et al., 2020; Nenninger et al., 2025) described in this study was kindly provided by Marian Bienstein (Institute of Biotechnology, RWTH Aachen University). We used the coding region of the mature CueO lacking the N-terminal periplasmic secretion signal (GenBank: EDU66368.1) and fused it to the MacHis adhesion-promoting peptide (GenBank: P34084.1) via an EA-linker coupled to a Strep-tag-II at its N-terminus (CueO-MacHis itself has no GenBank ID). To facilitate quantification, we also included a His-tag at the C-terminus of the EA-linker (**Figure 1A**). The coding region of the modified CueO-MacHis was codon-optimized for *N. tabacum* using GeneOptimizer (Eurofins Genomics, Ebersberg, Germany). This software offers codon optimization for *N. tabacum* but not for *N. benthamiana*; however, the species are in the same genus and share highly similar codon usage patterns. For cloning, the restriction sites EcoRI, plus the 5′-untranslated region (UTR) of the chalcone synthetase (CHS) gene (Reimold et al., 1983), and NcoI were added before the first amino acid of the mature protein such that the NcoI restriction site also provided the start codon. Furthermore, an XhoI restriction site was introduced directly after the stop codon. Eurofins Genomics delivered this sequence in the plasmid pEX-K248. The modified CueO-MacHis sequence was transferred to the vector pTRAc-rfp-H (for cytosolic expression without an additional signal peptide) using EcoRI and XhoI. It was also transferred using NcoI and XhoI to pTRAc-rfp-TPH (for chloroplast expression via translational fusion to the N-terminal transit peptide of the RuBisCO small subunit), pTRAc-rfp-ERH (for expression in the endoplasmic reticulum (ER) via translational fusion to the N-terminal ER-targeting peptide of the heavy chain of the monoclonal antibody mAb24 and the C-terminal ER retention signal SEKDEL), and pTRAc-rfp-AH (for apoplastic expression via translational fusion to the N-terminal mAb24 targeting peptide only) (Maclean et al., 2007). This enabled constitutive overexpression driven by the cauliflower mosaic virus (CaMV) 35S promoter (p35S, with a double enhancer) and terminator, including the 3′-UTR (t35S) (Odell et al., 1985) (**Figure 1B**). The final vectors were verified by in-house sequencing and transferred to *Agrobacterium tumefaciens* strain GV3101::pMP90RK (Koncz and Schell, 1986; Hellens et al., 2000) by electroporation, followed by selection on medium containing 25 mg/L rifampicin (resistance encoded on the bacterial chromosome), 25 mg/L kanamycin (resistance encoded on the pMP90RK helper plasmid), and 50 mg/L carbenicillin (resistance encoded on the pTRAc plasmid).

### Transient transformation of *Nicotiana benthamiana*

As described elsewhere (Nausch and Broer, 2017; Hou et al., 2022), *N. benthamiana* plants were grown in the greenhouse until the age of 2–8 weeks (as specified in the Results section) in peat soil (Stender, Schermbeck, Germany) and fertilized with 0.1% (m/v) Ferty 2 Mega (Nitsch & Sohn, Kreuztal, Germany) without CuSO_4_ supplementation. Additional illumination of 140 µmol/(s*m^2^) ensured a 16-h photoperiod. *A. tumefaciens* recombinant strains were seeded from glycerol stocks and cultivated in yeast extract broth (YEB) containing antibiotics as described above for 22–26 h at 28 °C, with shaking at 150 rpm in 100-mL Erlenmeyer shake flasks (Schott, Mainz, Germany) with a 10-mL filling volume. The pre-culture was then used to inoculate either 1-L Erlenmeyer shake flasks with baffles (Schott, Mainz, Germany) with a 100-mL filling volume (small-scale expression) or 2.5-L Ultra Yield Thomson flasks (Thomson Instrument Company, Carlsbad, USA) with a 500-mL filling volume (large-scale expression) in an orbital shaker as the main culture. After another 22–26 h of cultivation, the bacteria were centrifuged (5,000 × g, 15 min, 22 °C), and the pellet was resuspended in infiltration buffer (10 mM MES, 10 mM MgCl_2_, 0.2 mM acetosyringone, 0.2% Silwet Gold, pH 5.6) and adjusted to a final OD_600nm_ of 0.1–1.0 (as described in the Results). For vacuum infiltration, we submerged inverted *N. benthamiana* plants in the infiltration suspension and applied a vacuum of 100 mbar for 2 min in a desiccator. The plants were then returned to normal growth conditions and incubated in the dark for 12–16 h before illumination was switched on. The *N. benthamiana* leaf material was harvested 3–7 days post infiltration (dpi) (as specified in the Results). For each condition tested, at least three plants were pooled and stored at –80 °C for further processing.

### Preparation of the crude extract

For small-scale protein extraction, 5-g aliquots of frozen leaf material (at least a pool of three identically treated plants) were sampled in 50-mL Falcon tubes and homogenized at a ratio of 1:3 (m/v) with 15 mL Tris extraction buffer (50 mM Tris, 500 mM NaCl, pH 8.0) with or without cOmplete protease inhibitor cocktail (Roche, Basel, Switzerland) or 4 mM CuSO_4_ (as specified in the Results) using an Ultra Turrax T25 disperser (IKA-Werke, Staufen, Germany) at 24,000 rpm, 4 °C for 3 min. The homogenate was then centrifuged at 8,000 × g, 4 °C for 15 min, and the supernatant transferred to a new 50-mL Falcon tube. We analyzed the accumulation of recombinant protein in the insoluble fraction by resuspending the cell debris from the homogenate of the centrifugation step in NuPage Reducing Sample Buffer (Thermo Fisher Scientific, Waltham, USA) at a ratio of 1:3 (m/v). This was denatured at 90 °C for 10 min similarly to the preparation of the clarified crude extracts for Western blot analysis (see below).

For preparative-scale protein extraction, 100 g to 1 kg of frozen leaf material was homogenized three times in Tris extraction buffer at a ratio of 1:3 (m/v) using a ProBlend 6 3D mixer (Philips, Amsterdam, Netherlands) at 35,000 rpm for 30 s with 30-s breaks. The homogenate was then clarified either by two rounds of centrifugation at 8,000 × g, 4 °C for 30 min and 38,000 × g, 4 °C for 60 min, or alternatively by bag filtration using a BP420 bag filter (Fuhr, Klein-Winternheim, Germany) and depth filtration using either the PDH11 (K700 + V100 filter, with filter aids) or PDHB (K700 + Bio20 filter, without filter aids) (Pall Corporation, New York, USA). For subsequent chromatography, we filter-sterilized (0.45 µm + 0.22 µm) the clarified supernatant using a Sartopore 2 Capsule (Sartorius, Göttingen, Germany).

### Clarification of the crude extract by heat and pH precipitation

Initial extractions were conducted as described above by homogenization in Tris extraction buffer at a ratio of 1:3 (m/v) and centrifugation at 8,000 × g, 4 °C for 15–30 min. The extract was then stirred (100 rpm) in a pre-warmed water bath at 20 °C–70 °C (as specified in the Results) for 5 min (effective temperature in the extract measured using a temperature probe) and then cooled to 4 °C in an ice bath. The pH of the extract was then adjusted to 4.0–9.0 (as specified in the Results) by adding either 10 M HCl or 10 M NaCl and incubating for 5 min at the new pH (effective pH in the extract measured using a pH probe). The pH was then re-adjusted to 8.0 for immobilized metal affinity chromatography and anion exchange (AEX) chromatograhy or 5.0 for cation exchange (CEX) chromatography, followed by a centrifugation at 38,000 × g, 4 °C for 60 min to separate the precipitated proteins from the supernatant. IMAC must come first, followed by AEX and CEX. For subsequent chromatography, we filter-sterilized the supernatant as described above.

### Chromatography

Chromatography steps were carried out using an ÄKTA pure system (Cytiva, Marlborough, USA) at a flow rate of 1 mL/min for all experiments. For IMAC, we employed Chelating Sepharose Fast Flow resin (Cytiva) charged with Ni^2+^ ions. After equilibration with 10 column volumes (CV) of extraction buffer, the filter-sterilized extract was loaded (variable CV), and the column then washed with 4 CV of extraction buffer. Finally, the His-tagged target protein was eluted at a 10-CV gradient to 100% elution buffer (50 mM Tris, 500 mM NaCl, and 300 mM imidazole, pH 7.6) while collecting 2 mL fractions.

For AEX, we used a low-salt, alkaline Tris extraction buffer without sodium chloride at pH 9.0 (20 mM Tris, 4 mM CuSO_4_, pH 9.0, as specified in the Results). This was adjusted to pH 8.0 after heat treatment, diluted 1:2 (v/v) with MilliQ water to ensure the conductivity was below 5.0 mS/cm, and the extract then applied to a Q Sepharose Fast Flow resin (Cytiva). Equilibration and washing followed the IMAC method with 10 and 4 CV of extraction buffer at pH 8.0, whereas elution was achieved in a 10-CV gradient of extraction buffer with sodium chloride also adjusted to pH 8.0 (20 mM Tris, 0–500 mM NaCl, 4 mM CuSO_4_, pH 8.0, as specified in the Results). CEX differed only in using a low-salt, acidic extraction Tris buffer adjusted to pH 4.0, which was re-adjusted to pH 5.0 after heat treatment. Similarly, equilibration and washing used the same extraction buffer at pH 5.0, and elution used the same buffer with sodium chloride at pH 8.0 as described for AEX.

### Diafiltration

For small-scale diafiltration (DF) (without concentration), we used Vivaspin 15R centrifugal concentrators with a polyethersulfone (PES) membrane and a molecular weight cut off (MWCO) of 30 or 10 kDa (Sartorius) for centrifugation at 4,000 × g (as specified in the Results) for exchange into a neutral Tris buffer (20 mM Tris, pH 8.0) according to the manufacturer’s guidelines. The sample was concentrated to one-tenth of the initial volume and washed twice with the initial load volume but using the final buffer, before dissolving the retentate on the membrane in the final buffer at one-tenth of the initial volume.

For preparative-scale DF (with concentration) based on tangential flow filtration (TFF), we used the Sartorius Sartoflow Slice 200 Benchtop Crossflow System equipped with a Sartocon Slice 200 Regenerated Cellulose (RC) membrane (MWCO of 10 kDa) for buffer exchange into a neutral Tris buffer (20 mM Tris, pH 8.0), at a transmembrane pressure of 0.5 bar. Similar to the small-scale DF process, the sample was first concentrated to one-tenth of the initial volume, then washed with the initial load volume of the target buffer, and finally the retentate was resuspended to one-tenth of the initial volume.

### Protein quantification, dot blot, Western blot and densitometry

Protein quantification, dot blot, Western blot, and densitometry were carried out as previously described (Hou et al., 2022). The total soluble protein (TSP) concentration in extracts and supernatants was quantified using the Pierce Coomassie Protein Assay-Kit (Thermo Fisher Scientific). For dot blot quantification of native protein in the crude extract, each 100 µg of TSP and 1:2, 1:5, 1:10, 1:25, 1:50, and 1:100 dilutions thereof were directly applied to a nitrocellulose membrane, dried at 22 °C–24 °C for 30 min, and then treated as described below for Western blots. For Western blot analysis and Coomassie staining of the denatured protein, each 10 µL of extract was mixed with NuPage Reducing Sample Buffer (Thermo Fisher Scientific), denatured at 80 °C for 10 min, and separated on NuPAGE 4%–12% (m/v) Bis-Tris protein gels (Thermo Fisher Scientific) at 200 V and 22 °C–24 °C for 35 min. For Coomassie staining, the gels were stained in SimplyBlue SafeStain (Thermo Fisher Scientific) for 30 min. For Western blots, separated proteins were transferred to Amersham Protran nitrocellulose membranes (VWR, Radnor, USA) at 12 V and 22 °C–24 °C for 150 min using the XCell SureLock Mini-Cell and XCell II Blot Module (Thermo Fisher Scientific). After blocking for 1 h at 22 °C–24 °C with PBST (10 mM Na_2_HPO_4_, 1.8 NaH_2_PO_4_, 137 mM NaCl, 2.7 mM KCl, 0.5% (m/v) Tween-20, pH 7.2) supplemented with 5% (m/v) skimmed milk, the membrane was probed at 22 °C–24 °C for 90 min with rabbit polyclonal anti-His primary antibody (Cat. A00174; GenScript, Nanjing, China) diluted 1:5,000 in PBST. After three washes in PBST at 22 °C–24 °C for 5 min, the membranes were probed with alkaline phosphatase (AP)-conjugated goat anti-rabbit IgG polyclonal secondary antibody (Cat. 111-055-045; Jackson ImmunoResearch Laboratories, Baltimore Pike, USA) at a dilution of 1:5,000 in PBST for 90 min at 22 °C–24 °C. After three further washes with PBST at 22 °C–24 °C for 5 min, the membranes were stained with NBT/BCIP at 22 °C–24 °C for 30 min, and the reaction was then stopped by washing in MilliQ water. Densitometric analysis was carried out using AIDA Image Analysis software (Elysia-raytest, Straubenhardt, Germany).

We calculated the proportion of intact CueO-MacHis relative to the total amount of detected CueO-MacHis (intact protein plus degradation products) as follows:


Intact CueO‐MacHis (%)=(CueO‐MacHis full‐length [mg] / total detected CueO‐MacHis [mg])×100

where the amounts of full-length CueO-MacHis and total detected CueO-MacHis were determined by densitometry based on the corresponding Western blot signals.

CueO-MacHis protein purity was calculated as the ratio of the amount of CueO-MacHis, determined by densitometry based on the corresponding Western and dot blot signals using purified DsRed as a calibration standard, to the TSP, determined by the Pierce Coomassie Protein Assay-Kit (Thermo Fisher Scientific), as follows:


Purity (%)=(CueO‐MacHis [mg] /TSP [mg])×100

The step recovery of CueO-MacHis during each downstream step was calculated as follows:


Step recovery (%)=(CueO‐MacHis after process step [mg] / CueO‐MacHis before process step [mg])×100

The overall recovery of the downstream process was calculated as follows:


Overall recovery (%)=(CueO‐MacHis after process step [mg] / CueO‐MacHis in the crude extract [mg])×100

### Enzyme activity assay

The activity of CueO-MacHis was analyzed using a protocol provided by SeSam-Biotech (kindly provided by Francisca Contreras Leiva). Each 10 µL of purified enzyme with a defined concentration was added to 140 µL assay buffer (100 mM citric acid, 100 mM Na_2_HPO_4_, 1 mM CuSO_4_, 2 mM ABTS, pH 3.0), and the absorbance at 420 nm (A_420nm_) measured in kinetic mode at 1-min intervals for 10 min. We calculated the enzyme activity using the linear range of the absorbance increase according to the validated SeSaM-Biotech protocol, multiplied with an assay-specific conversion factor that accounts for the extinction coefficient of ABTS under the specified assay conditions, reaction volume, and optical path length:


Specific activity (U/mg)=(ΔA420/min)×0.948×DF

where DF is the dilution factor of the enzyme sample. The specific enzyme activity (U/mg) was calculated by dividing the enzyme activity (U/mL) by the corresponding CueO-MacHis concentration.

### Statistical methods

Statistical analysis was carried out using Origin 2020b (OriginLab, Northampton, USA). Data normality was assessed using the Shapiro–Wilk test, and homogeneity of variances assessed using Brown–Forsythe test. For normally distributed data, statistical significance was analyzed by one-way analysis of variance (ANOVA) followed by Bonferroni’s post-hoc test. For non-normally distributed data, we applied a Kruskal-Wallis ANOVA. In all cases, *p*< 0.05 was considered statistically significant. For experiments with fewer than three biological replicates (n < 3), statistical analysis was not performed because these preparative-scale proof-of-concept experiments were not set up for statistical evaluation.

DoE experiments were set up and analyzed using Design-Expert 13 (Stat-Ease, Minneapolis, USA).

## Results

### Bacterial CueO-MacHis is efficiently produced in chloroplasts at high yields

**Figure 2:**
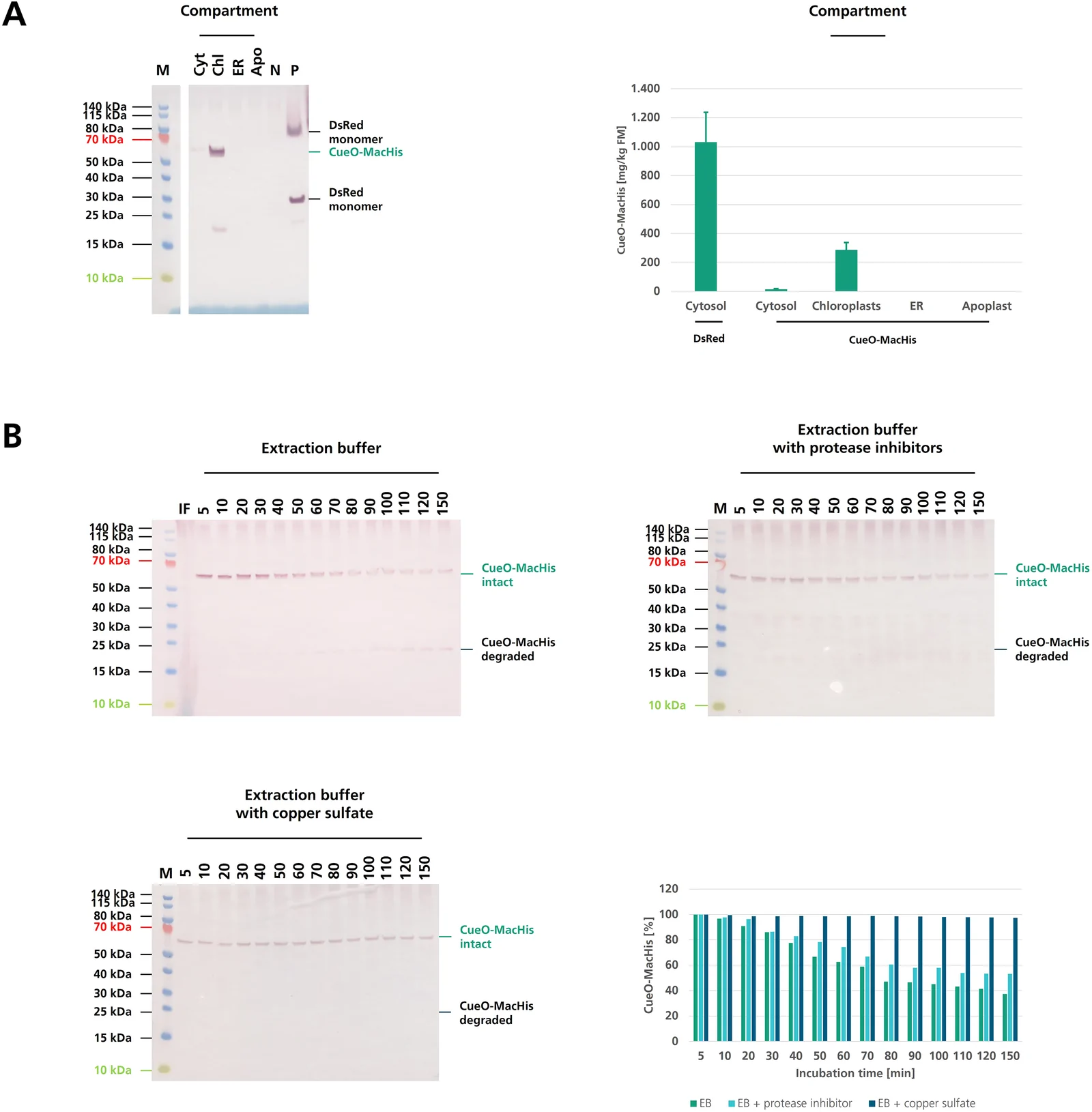
Compartment-specific accumulation and stability of the modified CueO-MacHis produced by transient transformation of *N. benthamiana*. **(A)** Accumulation of recombinant CueO-MacHis in different plant cell compartments: Cyt, cytosol; Chl, chloroplasts; ER, endoplasmic reticulum; Apo, apoplast; N, mock-infiltrated plants; P, plants infiltrated with the pTRAc vector expressing cytosolic DsRed. **(B)** Stability of recombinant CueO-MacHis in different extraction buffers: IF, insoluble fraction; EB, Tris extraction buffer. Protease inhibitor, cOmplete protease inhibitor cocktail (Roche, Basel, Switzerland); copper sulfate, 4 mM CuSO_4_. M, PageRuler (Thermo Fisher Scientific). Columns and error bars represent the mean and standard deviation. n_b_ = 3 **(A)** and n_b_ = 1 **(B)**.

In the initial screening, we expressed the engineered bacterial CueO-MacHis in different plant cell compartments, namely, the cytosol, chloroplasts, ER, and apoplast, which confer different redox environments essential for correct folding and copper chelation as well as contain different sets of proteases that may affect protein stability and accumulation. Using the standard settings for transient expression in *N. benthamiana* (6-week-old plants, *A. tumefaciens* OD_600nm_ = 0.4, and leaf harvest at 5 dpi), no soluble recombinant protein was detected when targeted to the ER or apoplast, and only low amounts (16 ± 4 mg/kg FM) were found in the cytosol. In contrast, CueO-MacHis levels reached 286 ± 35 mg/kg FM when targeted to chloroplasts, nearly a quarter of the amount of the unrelated recombinant fluorescent protein DsRed (1,031 ± 186 mg/kg FM), which served as an internal quantitative reference protein for comparison (**Figure 2A**). In addition to the expected product of ~62 kDa, we detected a faint fragment at ~22 kDa that indicated partial degradation of CueO-MacHis. We also investigated whether recombinant CueO-MacHis accumulated as insoluble protein in the cell debris but did not find it, suggesting that the product was largely soluble (**Supplementary Figure 1**).

To determine whether the soluble, chloroplast-targeted CueO-MacHis degraded *in planta* before extraction or in the extract after lysis, we prepared crude extracts and incubated the samples for 5–150 min as a time series (**Figure 2B**). Immediately after extract preparation (at 5 min), 99% of CueO-MacHis was intact, decreasing to 60% after 150 min. Moreover, the amount of both intact and degraded protein declined by 40%, which may be explained by the formation of an additional ~40 kDa split product (without His-tag) that could not be detected by Western blot. Hence, the recombinant CueO-MacHis remained intact when targeted to chloroplasts but degraded in the crude extract of lysed cells. To determine whether this degradation was caused by proteases, we added a protease inhibitor cocktail to the extraction buffer, but this only marginally reduced CueO-MacHis degradation compared with the extraction buffer control (**Figure 2B**). Consequently, the degradation of the recombinant CueO-MacHis protein may reflect intrinsic instability, perhaps due to incomplete copper incorporation. CueO chelates multiple copper ions, and CuSO_4_ is therefore added to the cultivation medium when CueO is produced in microbes (Novoa Henríquez, 2019; Nenninger et al., 2025). Using an extraction buffer containing CuSO_4_ instead of the protease inhibitor cocktail, no degradation product was observed in the extract after 150 min, and the total amount of CueO-MacHis remained nearly constant, with a final yield of 97% at 150 min compared to the initial amount found at 5 min (**Figure 2B**). Supplementation with CuSO_4_ thus appeared essential for stabilizing CueO-MacHis during extraction and purification. Although the stabilizing effect of CuSO_4_ was clearly demonstrated in this study, the extent of copper incorporation into the recombinant CueO-MacHis protein was not investigated in detail. Future studies, including quantitative copper determination, may provide further insights into the relationship between copper loading and protein stability. Moreover, because the purpose of this experiment was to evaluate the individual effects of the protease inhibitor cocktail and CuSO_4_ on CueO-MacHis recovery, and because CuSO_4_ alone efficiently stabilized the enzyme, we did not test both additives in combination.

**Figure 3:**
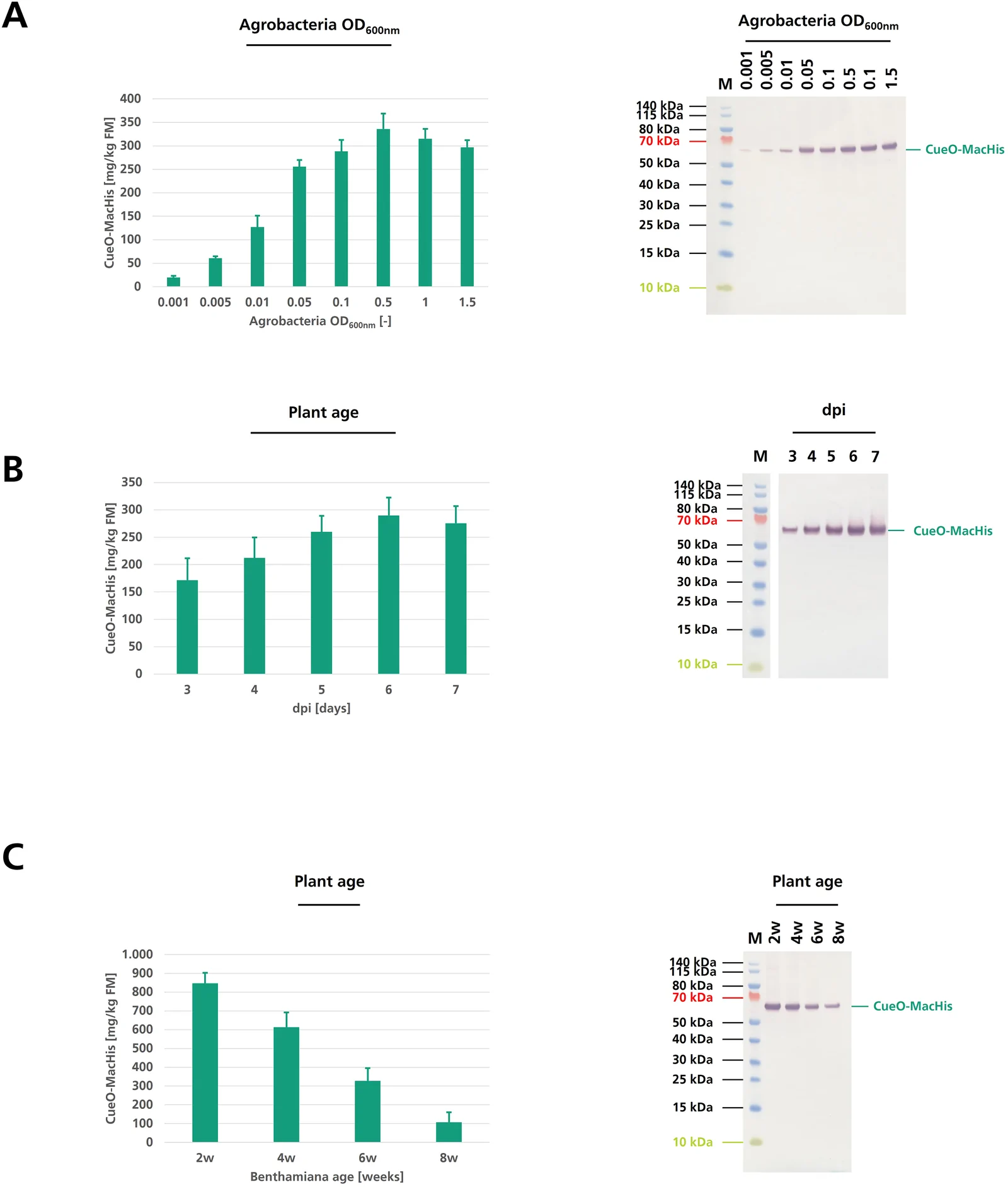
Optimization of the expression conditions for the modified CueO-MacHis produced by transient transformation of *N. benthamiana*. Accumulation of recombinant CueO-MacHis depending on **(A)** the infiltration density of *Agrobacterium*, **(B)** harvest day, and **(C)** plant age. OD_600nm_, optical density at 600nm; dpi, days post infiltration; w, week. M, PageRuler (Thermo Fisher Scientific). Columns and error bars represent the mean and standard deviation. n_b_ = 3 **(A–C)**.

To further optimize the yield of chloroplast-targeted CueO-MacHis, we analyzed the age of the *N. benthamiana* plants, *Agrobacterium* OD_600nm_, and harvest date after infiltration. Varying the *Agrobacterium* OD_600nm_ in the range from 0.1 to 1.0 showed that similar accumulation was achieved at OD_600nm_ 0.4–1.0, whereas lower values resulted in a statistically non-significant decline in concentration. We therefore extended the investigated range to 0.005–1.5 (**Figure 3A**). In this experiment, the OD_600nm_ range of 0.1–1.0 achieved similar yields to those obtained at the optimum of OD_600nm_ = 0.5, but yields dropped to 76 ± 3% at OD_600nm_ = 0.05 and 38 ± 11% at OD_600nm_ = 0.01. This suggests robust CueO-MacHis expression at OD_600nm_ > 0.1. The impact of the harvest date (3–7 dpi) was more pronounced (**Figure 3B**). CueO-MacHis accumulation increased continuously from 3 to 6 dpi and then stagnated at 7 dpi, demonstrating that the protein yield was influenced by protein synthesis rather than degradation. Analysis of *N. benthamiana* plants 2–8 weeks old revealed that increasing plant age decreased the relative accumulation of CueO-MacHis per kg FM: 846 ± 57 mg/kg FM (2 w), 613 ± 79 mg/kg FM (4 w), 328 ± 67 mg/kg FM (6 w), and 108 ± 54 mg/kg FM (8 w) (**Figure 3C**).

**Figure 4:**
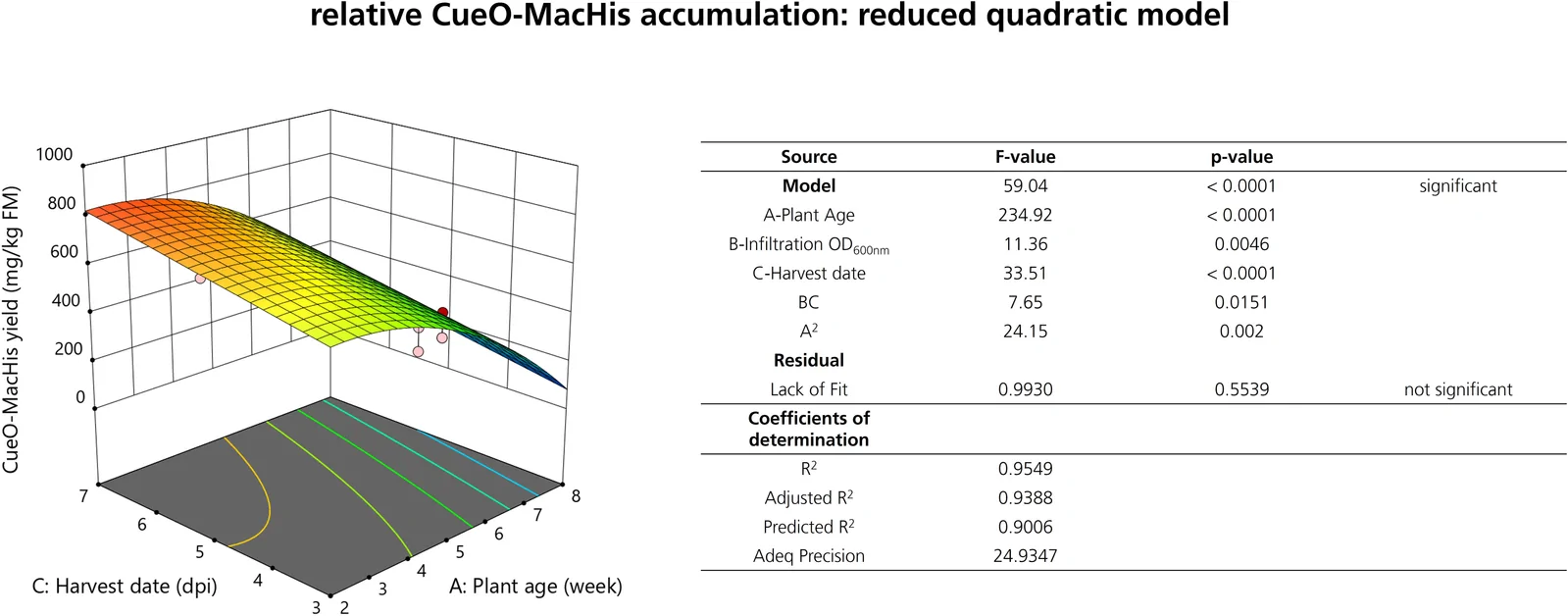
DoE-based optimization of the expression conditions of the modified CueO-MacHis produced by transient transformation of *N. benthamiana*. DoE-modeled impact of plant age, *Agrobacterium* OD_600nm_, and harvest date on relative CueO-MacHis accumulation. DoE, design of experiments. n_b_, not applicable.

Next, we used a DoE approach to optimize all three parameters simultaneously and identify the optimal trade-off. Because the selected parameters were already known to have an impact, we used a response surface optimal design with I-optimality, which is particularly suitable for prediction (instead of description) purposes and designed a quadratic model with 20 different factor combinations (runs) analyzing the effects of (A) plant age, (B) *Agrobacterium* OD_600nm_, and (C) harvest date, together with the factor interactions AB, AC, BC, A^2^, B^2^, and C^2^. This DoE can identify an effect that is 2.0 times the standard deviation of the replicates with a probability of 99%, 1.5 times the standard deviation with a probability of 89%, and 1.2 times the standard deviation with a probability of 51%. We selected the relative CueO-MacHis accumulation per biomass as the output parameter or response (**Figure 4**). A significant reduced quadratic model was obtained for relative CueO-MacHis accumulation. This model demonstrated a non-significant lack of fit, showing that the difference between the measured values and those predicted by the model for the same data point lies within the range of the standard deviation between replicates (i.e., the difference between the values of identical experiments). The reduced quadratic model included all three factors (A), (B), and (C), together with the interaction between *Agrobacterium* OD_600nm_ and harvest date (BC) and the quadratic interaction of plant age (A^2^) as significant factors. As indicated by the R^2^ of 0.96, the adjusted R^2^ of 0.94, and the predicted R^2^ of 0.90 for model quality, the DoE model fitted the data points very well and is suitable for optimum prediction. Based on the F-value, the age of the *N. benthamiana* plants used for infiltration had the strongest impact on relative CueO-MacHis accumulation (F = 234.92), whereas the impact of harvest day was rather moderate (F = 33.51) and that of *Agrobacterium* OD_600nm_ minor (F = 11.36). The impact of factor interactions BC (F = 7.65) and A^2^ (F = 24.15) was also minor, although the plant age had a stronger effect. Accordingly, numerical optimization recommended a plant age of 2 weeks, an *Agrobacterium* OD_600nm_ of 0.4, and a harvest day of 7 dpi, further supporting the assumption that the transgene expression cassette is strongly expressed and that the recombinant CueO-MacHis protein is stable, suggesting that the biosynthetic capacity of the *N. benthamiana* host plant is the limiting factor. And young plants with developing leaves contain a higher proportion of proliferating cells with high biosynthetic activity than older plants with mature cells and reduced biosynthetic activity.

Summarizing the work described so far, CueO-MacHis appeared to be a strongly expressed and stable recombinant protein when targeted to chloroplasts. The average yield was 286 ± 35 mg/kg FM when using 6-week-old *N. benthamiana* plants (the standard plant age for large-scale transient expression) but could be increased to 846 ± 57 mg/kg FM when 2-week-old plants were used.

### CueO-MacHis is effectively purified using a simple and economical protocol based on heat and/or pH treatment followed by AEX chromatography

Following the successful accumulation of recombinant CueO-MacHis in chloroplasts, we aimed to establish and systematically optimize a simple and scalable DSP strategy comprising biomass extraction, clarification, and final DF. The intracellular production of recombinant CueO-MacHis within chloroplasts necessitates tissue lysis to release the target protein, which generates abundant solid particles and, more importantly, soluble HCPs and secondray metabolites that need to be removed (Wilken and Nikolov, 2012; Dixon et al., 2018). Insoluble solid particles can easily be separated by centrifugation or filtration, but the removal of soluble HCPs and secondary metabolites is more challenging (Buyel et al., 2015). Orthogonal chromatography steps, such as affinity chromatography (AC) and ion-exchange chromatography (IEX), are usually required but are typically the most expensive DSP steps (approximately 50% of total DSP costs) and should therefore be minimized (Kelada et al., 2021). Given that CueO-MacHis is both temperature stable (up to 70 °C) and pH stable (4.0–9.0) (Novoa Henríquez, 2019; Nenninger et al., 2025), a combined temperature and pH treatment should eliminate the majority of HCPs during extraction, as previously demonstrated for other stable proteins produced in plants (Menzel et al., 2016; Opdensteinen et al., 2021). The remaining secondary metabolites can then be cleared in a single chromatography step.

**Figure 5:**
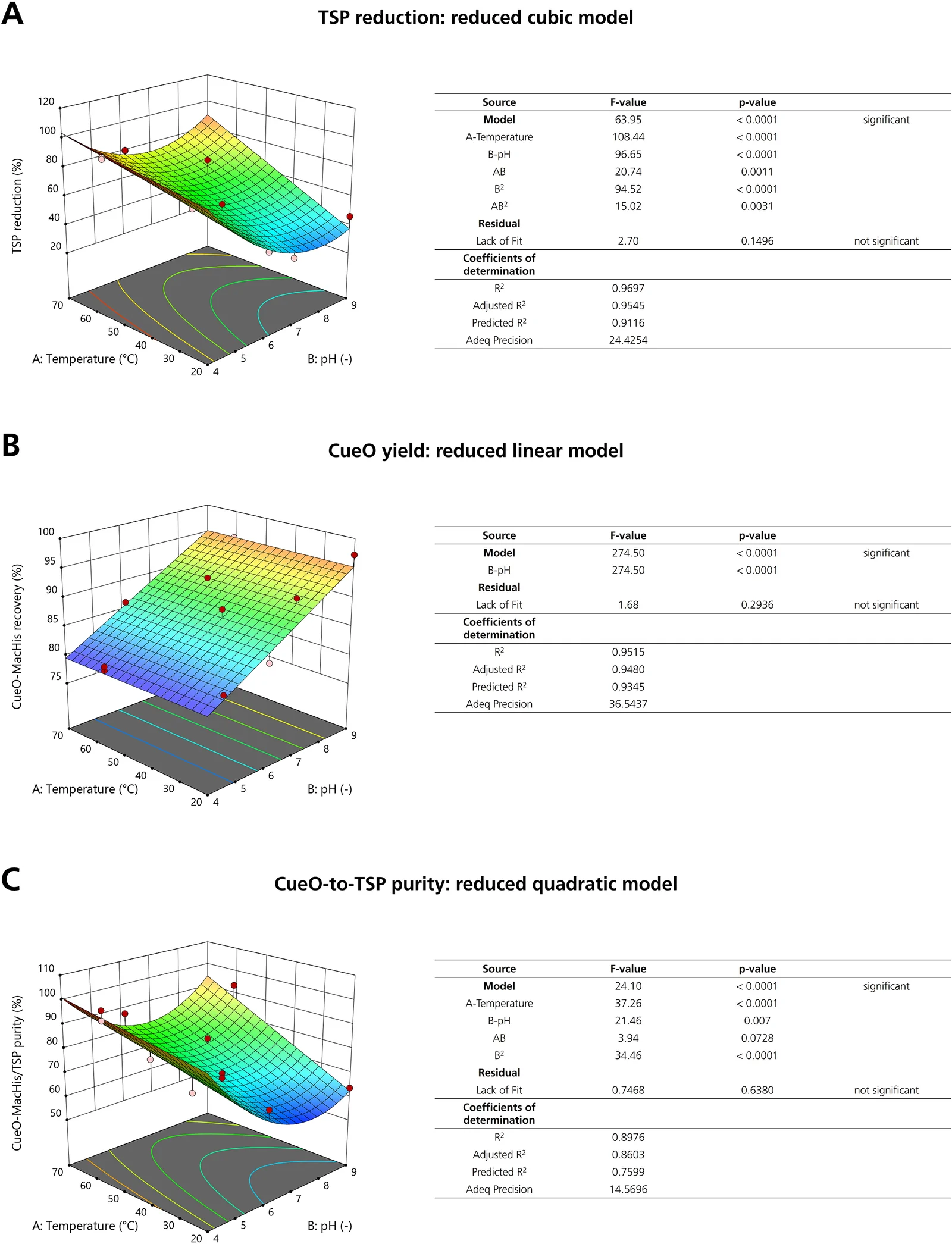
DoE-based optimization of the extraction conditions of the modified CueO-MacHis produced by transient transformation of *N. benthamiana*. DoE modeled temperature (20 °C– 70 °C) and pH (4.0–9.0) effects on **(A)** reduction of TSP, **(B)** recovery of CueO-MacHis, and **(C)** CueO-MacHis-to-TSP purity. DoE, design of experiments; TSP, total soluble protein. n_b_ = not applicable.

We used a DoE to investigate whether a combined temperature and pH treatment can be employed to separate CueO-MacHis from plant HCPs, allowing us to evaluate the impact of the individual factors and their interactions. Similar to the DoE optimization of USP conditions, it is already known that temperature and pH have an impact, so we again selected a response surface optimal design with I-optimality for prediction and designed a quadratic model with 16 different factor combinations (runs) analyzing the impact of (A) temperature, (B) pH, and the factor interactions AB, A^2^, and B^2^. This DoE can identify an effect that is 2.0 times the standard deviation of the replicates with 99% probability, 1.5 times the standard deviation with 91% probability, and 1.2 times the standard deviation with 67% probability. The output parameters (responses) were TSP reduction, CueO-MacHis recovery, and CueO-to-TSP purity. For TSP reduction, a reduced cubic model was obtained (R^2^ = 0.97, adjusted R^2^ = 0.96, and predicted R^2^ = 0.91) in which both increasing the temperature to 70 °C and decreasing the pH to 4.0 substantially reduced TSP, and thus HCP levels, by approximately 85% (70 °C) and 95% (pH 4.0), respectively (**Figure 5A**), in agreement with previous studies (Menzel et al., 2016; Opdensteinen et al., 2021). For CueO recovery, a reduced linear model was built (R^2^ = 0.95, adjusted R^2^ = 0.95, and predicted R^2^ = 0.94) with only pH as a relevant factor, whereby recovery seemed to increase with increasing pH, with approximately 80% recovery at pH 4.0 and 95% at pH 9.0 (**Figure 5B**). Combining both output parameters, a CueO-to-TSP purity model was generated (R^2^ = 0.90, adjusted R^2^ = 0.86, predicted R^2^ = 0.76), which predicted that the highest purity would be obtained at either 70 °C and pH 4.0 (approximately 100%) or 70 °C and pH 9.0 (approximately 95%) (**Figure 5C**).

Nevertheless, numerical optimization to maximize TSP reduction, CueO-MacHis yield, and CueO-MacHis-to-TSP purity recommended 70 °C and pH 9.0 as optimal conditions, with a desirability score of 0.907. We validated the numerical optimization by combining the recommended temperature of 70 °C with either pH 9.0 or pH 4.0, using 22 °C as the reference condition (**Supplementary Figure 2**). The validation confirmed that both a temperature of 70 °C and a pH of 4.0 achieved purities greater than 80%, but that pH 4.0 also reduced CueO levels, indicating that the highest CueO yield and purity would be obtained with a temperature of 70 °C and a pH of 9.0.

Following initial extract clarification, the next step was purification. The modified CueO-MacHis is equipped with both a His-tag and a Strep-tag-II and can therefore be purified by AC (**Figure 1A**). The IMAC Sepharose for His-tag purification is substantially cheaper (€5,000–20,000/L) than the Strep-Tactin Sepharose for Strep-tag-II purification (€20,000–80,000/L), so we tested IMAC. However, when extracting the recombinant CueO-MacHis at 70 °C and pH 9.0 for subsequent IMAC purification, the IMAC step recovery was only 67%, which yielded in turn a low overall recovery of 67% compared to the load with 81% (i.e., the clarified extract after heat and pH precipitation, centrifugation, and filter-sterilization) (**Table 1**). Only minor amounts of CueO-MacHis were detected in the flow-through (3%) and wash fraction (0.05%), implying the incomplete elution of bound CueO-MacHis. This, in turn, suggests that a substantial fraction of CueO-MacHis is so tightly bound to the IMAC resin that the binding cannot be broken by imidazole. One potential explanation is that, in addition to the His-tag, the MacHis adhesion-promoting peptide contains His residues and accordingly also promotes adhesion to the IMAC resin that is not affected by imidazole. Moreover, when comparing the load and elution fractions, the purity remained nearly constant at 95% and 92%, respectively, thus reflecting concentration instead of purification. Hence, the only benefit of this step was volume reduction.

**Table 1:** Purification of CueO-MacHis using different chromatographic methods.

Process step	Volume	TSP		CueO-MacHis		Recovery		Purity
	Total	Concentration	Total	Concentration	Total	Step	Total	
	[mL]	[µg/mL]	[mg]	[µg/mL]	[mg]	[%]	[%]	[%]
IMAC – pH 9.0 and 70 °C								
Homogenate	2,000.00	561.37	1,122.74	76.29	152.59	100.00	100.00	13.59
Clarified extract I (centrifugation)	1,800.00	557.54	1,003.57	75.41	135.74	88.96	88.96	13.53
Clarified extract II (pH 9.0 and 70 °C)	1,800.00	79.70	143.47	74.56	134.20	98.86	87.95	93.54
Clarified extract III (centrifugation)	1,750.00	79.30	138.77	73.95	129.41	96.43	84.81	93.26
Load (filter sterilization)	1,700.00	77.05	130.98	72.91	123.95	95.78	81.23	94.63
Flow-through	1,700.00	3.09	5.25	2.86	4.86	96.08	78.04	94.71
Wash	7.50	24.14	0.19	6.45	0.05	99.96	78.01	94.82
Elution	15.00	6,244.07	93.66	5,746.70	86.20	67.40	56.49	92.03
AEX – pH 9.0 and 70 °C								
Homogenate	500.00	516.59	258.30	70.73	35.37	100.00	100.00	13.69
Clarified extract I (centrifugation)	450.00	513.59	231.12	70.70	31.82	88.96	88.96	13.77
Clarified extract II (pH 9.0 and 70 °C)	450.00	76.44	34.40	70.68	31.80	99.97	89.92	92.46
Clarified extract III (centrifugation)	440.00	76.23	33.54	70.65	31.08	97.74	87.89	92.67
Load (filter sterilization + conductivity adjustment)	800.00	42.44	33.95	36.74	29.39	94.55	83.10	86.56
Flow-through	800.00	0.64	0.51	0.00	0.00	100.00	83.10	87.88
Wash	5.00	0.14	0.00	0.00	0.00	100.00	83.10	87.88
Elution	53.00	554.71	29.40	507.96	26.92	86.87	76.12	91.57
AEX – pH 4.0								
Homogenate	500	605.31	302.66	77.80	38.90	100.00	100.00	12.85
Clarified extract I (centrifugation)	450	599.42	269.74	76.29	34.33	88.25	88.25	12.73
Clarified extract II (pH 4.0 and 22 °C)	450	70.24	31.61	67.83	30.52	88.91	78.47	96.57
Clarified extract III (centrifugation)	440	70.41	30.98	67.32	29.62	97.03	76.14	95.61
Load (filter sterilization + conductivity adjustment)	800	38.12	30.50	35.26	28.21	95.25	72.52	92.50
Flow-through	800	1.88	1.50	0.00	0.00	100.00	72.52	97.30
Wash	5	0.74	0.00	0.00	0.00	100.00	72.52	97.31
Elution	53	482.30	25.56	475.50	25.20	86.87	64.78	98.59
CEX – pH 4.0								
Homogenate	500.00	562.53	281.27	70.80	35.40	100.00	100.00	12.59
Clarified extract I (centrifugation)	450.00	560.17	252.08	70.54	31.74	89.67	89.67	12.59
Clarified extract II (pH 4.0 and 22 °C)	450.00	65.15	29.32	61.66	27.75	87.41	78.38	94.64
Clarified extract III (centrifugation)	440.00	64.80	28.51	61.40	27.02	97.37	84.80	94.75
Load (filter sterilization + conductivity adjustment)	800.00	34.26	27.41	32.17	25.74	95.27	72.71	93.92
Flow-through	800.00	25.93	20.74	25.38	20.30	21.12	15.36	81.59
Wash	5.00	0.19	0.00	0.00	0.00	100.00	15.36	81.60
Elution	53.00	125.01	6.63	6.63	5.82	47.01	16.45	87.88
DF (Regenerated Cellulose) – pH 9.0 and 70 °C								
Homogenate	500.00	504.45	252.22	69.18	34.59	100.00	100.00	13.71
Clarified extract I (Miracloth)	450.00	502.21	226.00	68.81	30.96	89.51	89.51	13.70
Clarified extract II (pH 9.0 and 70 °C)	450.00	73.88	33.25	68.51	30.83	99.56	89.12	92.72
Clarified extract III (centrifugation)	440.00	73.49	32.33	68.26	30.03	97.43	86.83	92.89
[-]*								
DF retentate	36.00	797.45	28.71	715.94	25.77	85.81	74.51	89.78
DF retentate wash	36.00	747.36	26.91	653.07	23.51	91.22	67.97	87.38
DF retentate final	36.00	666.83	24.01	553.63	19.93	84.77	57.62	83.03

IMAC, immobilized metal affinity chromatography; AEX, anion exchange; CEX: cation exchange; DF, diafiltration; TSP, total soluble protein. *Diafiltration does not require filter sterilization of the clarified extract. n_b_ = 1.

Because CueO-MacHis was already highly pure in the load due to the temperature and pH treatment, we investigated whether it could be further purified and concentrated by IEX, as previously demonstrated for a recombinant bacterial xylanase as another technical enzyme produced in plant chloroplasts (Leelavathi et al., 2003). Both AEX and CEX Sepharose matrices are substantially cheaper (€1000–6000/L) than IMAC or Strep-Tactin Sepharose. Because CueO-MacHis can be extracted at either pH 9.0 or 4.0, we tested both AEX (pH 9.0) and CEX (pH 4.0) (**Table 1**). An alkaline Tris extraction buffer at pH 9.0 combined with a temperature of 70 °C, followed by AEX purification at pH 8.0, resulted in an AEX step recovery of 86% and a final overall recovery of 76% compared to the load with a recovery of 88%. The 86% AEX step recovery was therefore substantially higher than the IMAC step recovery of 67%, showing that AEX is more suitable than IMAC for CueO-MacHis purification. However, as described above for IMAC, enzyme purity only increased marginally (91% in the load and 92% in the eluate). We also investigated extraction under acidic conditions (pH 4.0 and temperature of 22 °C) followed by AEX purification at pH 8.0 (**Table 1**). This approach resulted in a lower final recovery (65%), mainly because recovery following acidic treatment in the load was only 75%, whereas AEX achieved a similar step recovery of 87% as before. The lower extraction efficiency via acidic treatment is consistent with the DoE results for the extraction conditions (**Figure 5**). When extracting under acidic conditions (pH 4.0 and temperature of 22 °C) followed by CEX purification at pH 5.0, 83% of the initial amount of CueO-MacHis was detected in the load with a purity of 95%, comparable to that obtained after extraction at 70 °C and pH 9.0 (**Table 1**). However, 77% of the loaded CueO-MacHis was found in the CEX flow-through, yielding a CEX step recovery of 22%, such that only 17% of the initial CueO-MacHis was recovered in the eluate. Visual inspection revealed a color change in the buffer and matrix, suggesting the copper ions were binding the negatively charged SP group of the CEX matrix and thus preventing the binding of CueO-MacHis (data not shown). Hence, when comparing IMAC, AEX, and CEX, AEX yielded the best purification performance, yielding a final recovery of 76% and a purity of 92%.

Nevertheless, the AEX step increased the concentration but not the purity of the recombinant CueO-MacHis, and this can also be achieved by TFF-based DF, which is considerably cheaper than chromatography resins, with costs of €500–2,500/m^2^. During initial DF tests, a low-protein-binding RC membrane with a MWCO of 30 or 10 kDa showed that the CueO-MacHis, with its calculated molecular mass of 62 kDa, passed through the 30 kDa MWCO membranes but was retained by the 10 kDa MWCO membrane (**Supplementary Figure 3**). Accordingly, we used the latter MWCO for all subsequent tests. TFF was less effective than AEX, resulting in a final recovery of 58% and a lower purity of 83% (**Table 1**). The low recovery can be attributed to the recoveries of 68%–75% in the individual DF steps, possibly due to CueO-MacHis, which precipitated but could not be redissolved and/or bound to the DF membrane and could not be eluted. Accordingly, the TFF step was outperformed by AEX in terms of both CueO-MacHis recovery and purity. AEX therefore seems to be the most suitable option for the purification of CueO-MacHis.

We also tested whether centrifugation for extract clarification could be replaced with a more scalable bag filtration (to remove coarse particles) and depth filtration (to remove fine particles) step (Buyel, 2026). The BP420 bag filter (composed of polypropylene needle felt, with a nominal pore size of 1–200 μm) is commonly used for initial clarification, followed by various depth filters with filter aids (composed of cellulose fibers and inorganic filter aids, such as diatomaceous earth or perlite) for secondary clarification. Typical filters with filter aids included the PDH11 (nominal pore size of 2–20 μm), PDH4 (nominal pore size of 0.5–15 μm), and PDF4 (nominal pore size of 0.5–10 μm), which are selected according to the particle load in the bag-filtered extract. However, even though combined BP420 bag filtration and PDH11 depth filtration yielded a similar purity to that of the load (91%), the recovery after filter sterilization was only 56% (**Table 2**). Because the main reason for the low recovery was the relatively high CueO-MacHis loss of 28% during depth filtration, and because this loss might have been associated with binding of the recombinant protein to the filter aids (Buyel, 2026), we also tested the depth filter PDHB without filter aids (nominal pore size 0.5–15 µm). This did not improve the recovery, which remained similar, with a step recovery of 69% for the depth filter and a total recovery of 53% in the load (**Table 2**). Accordingly, product recovery of the filtration-based clarification, at 53-56% in the load (**Table 2**), was inferior to that achieved by centrifugation-based clarification, at 81%–87% (**Table 1**, extractions at a temperature of 70 °C and pH 9.0). We therefore used centrifugation in all further experiments.

**Table 2:** Comparison of PDH11 and PDHB depth filters in terms of the CueO-MacHis recovery.

Process step	Volume	TSP		CueO-MacHis		Recovery		Purity
	Total	Concentration	Total	Concentration	Total	Step	Total	
	[mL]	[µg/mL]	[mg]	[µg/mL]	[mg]	[%]	[%]	[%]
PDH11 (with filter aids)								
Homogenate (bag filtration)	500.00	730.14	365.07	96.88	48.44	100.00	100.00	13.27
Clarified extract I (bag filtration)	440.00	682.22	300.18	91.75	40.37	83.34	83.34	13.45
Clarified extract II (pH 9.0, temp. 70 °C)	440.00	99.73	43.88	90.65	39.89	98.80	82.34	90.89
Clarified extract III (depth filtration)	425.00	74.55	31.68	67.67	28.76	72.11	59.37	90.78
Load (filter sterilization)	420.00	70.71	29.70	64.12	26.93	93.64	55.60	90.68
PDHB (without filter aids)								
Homogenate (bag filtration)	500.00	707.78	353.89	88.64	44.32	100.00	100.00	12.52
Clarified extract I (bag filtration)	440.00	676.78	297.78	83.78	36.86	83.18	83.18	12.38
Clarified extract II (pH 9.0, temp. 70 °C)	440.00	88.39	38.89	82.11	36.13	98.00	81.51	92.89
Clarified extract III (depth filtration)	425.00	64.13	27.26	58.89	25.03	69.28	56.47	91.83
Load (filter sterilization)	420.00	61.18	25.69	56.09	23.56	94.12	53.15	91.69

PDH11, K700 + V100 filter (nominal pore size 2–20 µm, with filter aids); PDHB, K700 + Bio20 filter (nominal pore size 0.5-15 µm, without filter aids). n_b_ = 1.

Regardless of the extraction and purification methods, the CueO-MacHis purified from plant extracts proved to be enzymatically active, averaging 7.22 ± 1.55 U/mg. This was slightly lower than that of its bacterial counterpart, which had an average of 9.34 ± 1.96 U/mg (**Table 3**).

**Table 3:** CueO-MacHis enzyme activity depending on the upstream production (USP) platform and downstream processing (DSP) protocol.

Expression platform	*E. coli*	*N. benthamiana*	*N. benthamiana*	*N. benthamiana*	*N. benthamiana*
Clarification	[-]	pH 9.0, 70 °C	pH 9.0, 70 °C	pH 4.0, 22 °C	pH 9.0, 70 °C
Purification	StrepTrap	IMAC	AEX	AEX	TFF
Activity [U/mg]	9.34 ± 1.96	7.22 ± 2.11	7.14 ± 2.17	7.32 ± 1.35	7.20 ± 1.55

StrepTrap: Strep-Tactin affinity chromatography; IMAC: immobilized metal affinity chromatography; AEX, anion exchange. *E. coli, purified CueO-MacHis provided by SeSam-Biotech and produced as described by Nenninger et al. (2025). n_b_ = 2-3.

Because CueO-MacHis activity could not be reliably quantified in crude plant extracts due to the low target protein concentration and the complex plant protein background, activity was measured only after purification.

Taken together, the expected molecular weight, specific anti-His immunodetection, and the characteristic laccase activity of the purified protein strongly support the identity of CueO-MacHis. Additional confirmation by LC-MS/MS or peptide mass fingerprinting would further strengthen the molecular characterization of the recombinant protein and may be considered in future studies.

## Discussion

In this study, we demonstrated that the engineered bacterial laccase CueO-MacHis can be produced with yields of 286 ± 35 mg/kg FM in a soluble form when targeted to chloroplasts, which provide a prokaryotic environment. Furthermore, we established a cost-efficient extraction and purification process based on either heat treatment at 70 °C and/or pH treatment at 4.0 combined with AEX, yielding a final recovery of 76% with a purity of 92%. The purified enzyme also proved to be enzymatically active, with an average specific activity of 7.22 ± 1.55 U/mg.

### Upstream production

The high accumulation of CueO-MacHis observed following chloroplast targeting is consistent with the prokaryotic origin of chloroplasts, whose protein folding and maturation machinery is evolutionarily more closely related to that of bacteria than to the eukaryotic cytosol or secretory pathway. This evolutionary relationship probably contributes to the efficient production and accumulation of bacterial proteins in chloroplasts. The yields in plants are higher than those achieved for the engineered CueO-MacHis produced in *E. coli*, which are in the range of 25–50 mg/L (Novoa Henríquez, 2019; Zhou et al., 2024; Nenninger et al., 2025). Notably, the cultivation medium was supplemented with CuSO_4,_ and thus we needed to test whether adding CuSO_4_ to the fertilizer would also boost accumulation in plants. Independently, the optimization of transient expression in *N. benthamiana* showed that the OD_600nm_ in the range 0.4–1.0 had only a minor influence, which indicates that transgene transcription and/or translation is extremely efficient in the CueO-MacHis expression cassette and that even low amounts of *Agrobacterium* and T-DNA are sufficient to exploit the maximum protein biosynthesis capacity of plant cells, so that a further increase in the OD_600nm_ does not have any boosting effect. Furthermore, high *Agrobacterium* OD_600nm_ values are known to be counterproductive by inducing plant defense responses and/or transcriptional gene silencing (TGS) and post-transcriptional gene silencing (PTGS), thus inhibiting (recombinant) protein biosynthesis (Beritza et al., 2024; Kopertekh, 2024). This is consistent with other studies analyzing the transient expression of model proteins such as GUS and GFP, where OD_600nm_ values of 0.1, 0.5, and 1.0 had no significant effect, whereas excessive ODs potentially caused deleterious effects (Wydro et al., 2006; Lindbo, 2007; Shamloul et al., 2014; Norkunas et al., 2018; Tang et al., 2026). In terms of the harvest date, the accumulation of CueO-MacHis peaked at 6 dpi. This may reflect the balance between transient expression/protein production and the intrinsic instability of CueO-MacHis (as shown above), both of which affect overall protein accumulation. Up to 5–6 dpi, protein expression and production appeared to exceed protein degradation, resulting in increased CueO-MacHis accumulation. However, at 6–7 dpi, protein degradation appears to exceed production, resulting in reduced yield. This agrees with previous reports showing that transient expression was highest at 3 dpi and protein accumulation peaked after 4–6 days, at least in the case of expression cassettes without viral replicon elements (Zischewski et al., 2016; Jansing and Buyel, 2019). Plant age had the most substantial impact on relative protein accumulation per unit biomass, reaching 846 ± 57 mg/kg FM (2 weeks), 613 ± 79 mg/kg FM (4 weeks), 328 ± 67 mg/kg FM (6 weeks), and 108 ± 54 mg/kg FM (8 weeks). Notably, optimization of the cultivation strategy increased the accumulation of recombinant CueO-MacHis from 286 ± 35 mg/kg FM under the standard transient expression conditions to a maximum of 846 ± 57 mg/kg FM in 2-week-old *N. benthamiana* plants, corresponding to an approximately threefold improvement. This demonstrates the considerable impact of systematic process optimization on recombinant protein accumulation and highlights that cultivation parameters alone can substantially influence recombinant protein yields in PMF. This also agrees with previous studies showing that the relative accumulation of the model protein GFP declined with the age of the *N. benthamiana* plants used for infiltration, whereas the biomass stagnated when plants started flowering, reflecting the switch from vegetative to generative development (Sheludko et al., 2007; Leuzinger et al., 2013; Saur et al., 2016; Dodds et al., 2023; Zhang et al., 2023). This is also evidenced by studies showing that young and growing leaves accumulated more recombinant protein than fully developed mature leaves, with a sharp drop in senescent leaves (Sheludko et al., 2007; Buyel and Fischer, 2012; Goulet et al., 2019). Nevertheless, when combined with the parallel increase in biomass of 10 ± 3 g FM/plant (2 weeks), 54 ± 15 g FM/plant (4 weeks), 108 ± 17 g FM/plant (6 weeks), and 132 ± 15 g FM/plant (8 weeks), the highest absolute CueO-MacHis yield of 35 ± 7 mg/plant was obtained at 6 weeks, compared to 33 ± 6 mg/plant (4 weeks), 14 ± 7 mg/plant (8 weeks), and 8 ± 1 mg/plant (2 weeks). We also calculated the relative yield per area and time (taking into account the increasing space demand per plant and prolonged batch cycles) and found that 60 ± 5 g/(m^2^*52 w) can be produced when *N. benthamiana* plants are grown for only 2 weeks, compared with 30 ± 6 g/(m^2^*52 w) for 4 weeks, 10 ± 2 g/(m^2^*52 w) for 6 weeks, and 2 ± 1 g/(m^2^*52 w) for 8 weeks.

Only the chloroplast-targeted CueO-MacHis protein accumulated to high levels (286 ± 35 mg/kg FM). We speculate that the neutral to alkaline pH of 7.8–8.0 (in the light) and 7.0–7.3 (in the dark) in the chloroplast stroma stabilizes the enzyme, given that several bacterial laccases from *Streptomyces*, *Pseudomonas,* and *Bacillus* species are more stable at higher pH values (Chauhan et al., 2017; Arregui et al., 2019; Liu et al., 2023). However, pH cannot be the only parameter affecting the protein accumulation because the cytosol also provides a neutral environment with a pH of 7.0–7.5, yet cytosolic CueO-MacHis yielded only 16 ± 4 mg/kg FM. Another potential advantage of chloroplasts is that the recombinant CueO-MacHis is protected from cytosolic proteases. To the best of our knowledge, Lac51 is the only other bacterial laccase expressed thus far in plants by either transient expression or stable transformation; all other plant-expressed laccases were fungal laccases, which differ substantially from the bacterial ones (van Eerde et al., 2022). When transiently expressed in the cytosol, the authors reported that the recombinant Lac51 seemed to be unstable, which agrees with our assumption that bacterial laccases are prone to degradation by eukaryotic proteases. It remains to be determined whether higher yields of Lac51 might be obtained by targeting Lac51 to chloroplasts. Nevertheless, the markedly higher accumulation of CueO-MacHis observed following chloroplast targeting indicates that this subcellular compartment is not merely a suitable expression site but may represent the key enabling factor for efficient production of the bacterial MCO CueO-MacHis in plants.

An alternative to the transient expression of chloroplast-targeted CueO-MacHis is the generation of stably transformed transplastomic plants. Yields of 1–5 g/kg have been reported for other bacterial proteins, even when plants were grown in the field (Oey et al., 2009a, b; Castiglia et al., 2016; Zhu et al., 2017; Schmidt et al., 2019). These high yields, on the one hand, reflect the high transgene copy number in transplastomic plants (100–200 copies of circular plasmid DNA) compared with transient expression (5–20 copies of T-DNA), and, on the other hand, reflect the efficient redirection of amino acids away from RuBisCO synthesis, which represents 50%–80% of TSP in plants (Bally et al., 2009, 2011; Schmidt et al., 2019). It remains to be determined whether this would also be suitable for recombinant bacterial CueO-MacHis. Plants producing up to 5 g/kg FM of the recombinant target protein generally show a retarded growth phenotype, but this might be mitigated by using inducible expression cassettes to uncouple growth from the production, as demonstrated for field-grown transgenic plants (Tusé et al., 2014; Kelada et al., 2021). Although we did not complete a TEA for the CueO-MacHis production process, and although the production costs are highly process-specific, previous studies on comparable plant-based production systems for technical enzymes show the potential economic competitiveness of PMF. For example, inducible expression of cellulase (another technical enzyme) in transgenic plants grown in open fields achieved accumulation levels of 4 g/kg FM, with total USP (without DSP) costs of $7/kg (€6/kg) compared with $11/kg (€9/kg) when produced by fungal fermentation in bioreactors (Tusé et al., 2014). The lower costs mainly reflect the 85% lower capital investment (because no bioreactors are needed) and the 30% lower actual production costs. This demonstrates the potential of using transgenic plants for the production of technical enzymes compared with established fermentation systems. Another study concerning production of the food protein thaumatin (accumulation level: 1.5 g/kg FM) reported USP costs of $72/kg (€62/kg) for the same inducible expression strategy in field-grown transgenic plants (Kelada et al., 2021), suggesting that production costs would be in the range of €10–80/kg. Most importantly, the latter study also calculated DSP costs of $499/kg (equivalent to €430/kg), representing approximately 85% of total production costs. Accordingly, the final costs of $571/kg (€492/kg) would be higher than the market price for technical laccases, which is currently $35–80/kg (€30–69/kg) (Brugnari et al., 2021; Coherent Market Insights, 2025). However, these prices relate to fungal enzymes such as *Trametes versicolor* laccase, and it is unclear whether the same costs can be achieved for bacterial laccases produced in bacteria, e.g., *E. coli*, or yeast, e.g., *P. pastoris* or *S. cerevisiae*.

### Downstream processing

Whereas fungal laccases are secreted into the cultivation medium and can easily be recovered from it, most recombinant proteins in plants are produced intracellularly (in the chloroplasts for our laccase), and plant tissue must be homogenized, releasing both insoluble particles and soluble HCPs, resulting in the need for multiple filtration and chromatography steps (Wilken and Nikolov, 2012; Dixon et al., 2018). However, the thermal and pH stability of CueO-MacHis enabled us to establish a simple and cost-efficient clarification process in which the HCPs were precipitated by combined heat and pH treatment (70 °C and pH 9.0 or pH 4.0 for 5 min). This, in turn, enabled the one-step removal of insoluble particles and precipitated HCPs in a single centrifugation step. Furthermore, centrifugation can be replaced by a more scalable depth filtration step. The depth filter step recovery of 69%–72% was within the range of 46%–88% previously reported for other recombinant proteins in plant extract feeds clarified by depth filtration (Kelada et al., 2021; Knödler et al., 2023; Knödler and Buyel, 2023; Ridgley et al., 2023; Opdensteinen and Buyel, 2024), achieving a final product recovery of 53-56%. Although this was inferior to centrifugation (81%–87%), filtration may still offer a more economical DSP given the substantially lower investment costs for equipment and greater scalability. This is particularly relevant for technical enzymes that are produced and processed in the ton scale of biomass and extract volumes in the range of 10,000–100,000 L (Tusé et al., 2014; Kelada et al., 2021; Buyel, 2026). Importantly, the combined temperature and pH step alone yielded a CueO-MacHis purity exceeding 90%, which may already be sufficient for the intended application as a technical enzyme for wastewater treatment, eliminating additional chromatography steps. For the abovementioned thaumatin protein, which was also purified using such a process, omitting the chromatography step would reduce DSP costs by approximately 50% from $499/kg to $258/kg (€222/kg) (Kelada et al., 2021).

We demonstrated that CueO-MacHis could be isolated from the clarified extract and concentrated by AEX, also exploiting the stability of CueO-MacHis at pH 9.0. AEX is substantially cheaper than conventional AC (e.g., AEX Q Sepharose is €1,000–6,000/L compared to IMAC Sepharose at €5,000–20,000/L or Strep-Tactin Sepharose at €20,000–80,000 €/L), resulting in significant DSP cost savings. This strategy is consistent with a study in which a xylanase technical enzyme for bioethanol production was produced and purified from plant biomass using the same approach (Leelavathi et al., 2003), in addition to the food protein thaumatin (Kelada et al., 2021). Given that none of the chromatography methods we tested increased the purity directly, reducing the volume instead, TFF might be used, given the significantly lower DF membrane costs of €500–2,500/m^2^. However, this reduced the overall recovery from 76% (AEX) to 58%. We used a more hydrophilic low-protein-binding RC as a DF membrane, so a more hydrophobic low-protein-binding cellulose acetate membrane might be tested in the future, as already described for the purification of CueO-MacHis (Novoa Henríquez, 2019). Nevertheless, in our study, TFF was outperformed in terms of both recovery and purity by AEX chromatography, so the latter remains the most suitable method for the purification of CueO-MacHis.

The CueO-MacHis purified from plant biomass proved to be enzymatically active, and the different extraction and purification methods did not seem to affect the specific activity (7.22 ± 1.55 U/mg), even though its activity was slightly lower than that of its bacterial counterpart (9.34 ± 1.96 U/mg) (**Table 3**). We speculate that this resulted from unwanted oxidization due to the polyphenols in the extract, given the absence of an antioxidant such as sodium bisulfite or polyvinyl polypyrrolidone in the copper-containing extraction buffer (Buyel, 2026), and this additive should be tested in the future. Another possibility is incomplete copper loading of CueO-MacHis, as CueO-MacHis was unstable in the extract buffer without CuSO_4_ supplementation. As discussed above, further quantitative copper determination studies may provide insights into the relationships between copper loading, protein stability, and enzymatic activity. Nevertheless, CueO-MacHis activity was in the same range as reported for other CueO enzymes (Nenninger et al., 2025) and can be used as a technical enzyme for wastewater treatment.

Although several process development experiments were conducted with n = 1 under preparative-scale conditions, the resulting data provide relevant engineering data for process development and scale-up. Future studies should validate these findings using replicated pilot-scale experiments and further scaling up.

Moreover, our study demonstrates the technical feasibility of producing and purifying an active bacterial laccase in plants at high yield; however, a comprehensive comparison of plant-based and microbial production platforms ultimately requires additional performance metrics beyond recombinant protein accumulation. Parameters such as biomass productivity, volumetric productivity, DSP efficiency, production costs, and overall process economics are needed to evaluate the industrial competitiveness of different manufacturing platforms. Comprehensive TEAs and LCAs based on comparable process data are beyond the scope of the present study, but are important next steps to assess the economic viability and environmental sustainability of PMF for the production of technical enzymes.

## Conclusion

We have demonstrated through systematic optimization of USP and DSP that recombinant bacterial enzymes for technical applications can potentially be produced in plants in an economically viable and more environmentally sustainable manner by exploiting the prokaryotic environment of chloroplasts. These findings broaden the application spectrum of PMF beyond biopharmaceuticals towards industrial biotechnology and provide the basis for future TEA, LCA, and industrial implementation.

## Data Availability

The raw data supporting the conclusions of this article will be made available by the authors, without undue reservation.
