# Supplementary material for: Plant chloroplasts enable high-yield and cost-effective production of the engineered bacterial enzyme CueO-MacHis as a technical protein via transient expression in Nicotiana benthamiana
Source: Front Plant Sci. 2026 Aug 19;17:1907380. doi: 10.3389/fpls.2026.1907380 (PMC13533953; doi:10.3389/fpls.2026.1907380)
Supplement: Supplementary file 1 [file DataSheet1.docx]

**Supplementary Figure 1: Accumulation of recombinant CueO-MacHis as soluble and insoluble protein.** Cyt: cytosol, Chl: chloroplasts, ER: endoplasmic reticulum, Apo: apoplast, M: PageRuler (Thermo Fisher Scientific), S: Soluble protein, I: Insoluble Protein.

**Supplementary Figure 2: Verification of the DoE-based optimization of extraction conditions.** DoE: Design-of-Experiment, M: PageRuler (Thermo Fisher Scientific).

| **Process step** | **Volume** | **TSP** | | **CueO-MacHis** | | **Recovery** | | **Purity** |
| --- | --- | --- | --- | --- | --- | --- | --- | --- |
|  | **total** | **conc.** | **total** | **conc.** | **total** | **step** | **total** |  |
| **[-]** | **[mL]** | **[µg/mL]** | **[mg]** | **[µg/mL]** | **[mg]** | **[%]** | **[%]** | **[%]** |
| **Vivaspin 30R** | | | | | | | | |
| **DF**  **load** | 15 | 567.64 | 8.52 | 522.4.3 | 7.84 | 100.00 | 100.00 | 92.03 |
| **DF**  **retentate** | 15 | 56.56 | 0.85 | 51.0 | 0.77 | 9.76 | 9.76 | 90.17 |
| **DF**  **permeate I** | 10 | 677.35 | 6.77 | 636.38 | 6.36 | 81.21 | 81.21 | 93.95 |
| **DF**  **permeate II** | 10 | 0.00 | 0.00 | 0.00 | 0.00 | 0.00 | 0.00 | 0.00 |
| **Vivaspin 15R** | | | | | | | | |
| **DF**  **load** | 15 | 605.76 | 0.09 | 553.58 | 8.30 | 100.00 | 100.00 | 91.39 |
| **DF**  **retentate** | 15 | 593.92 | 8.91 | 543.88 | 8.16 | 98.25 | 98.25 | 91.58 |
| **DF**  **permeate I** | 10 | 8.76 | 0.09 | 1.67 | 0.02 | 0.31 | 0.30 | 19.01 |
| **DF**  **permeate II** | 10 | 0.00 | 0.00 | 0.00 | 0.00 | 0.00 | 0.00 | 0.00 |

DF, diafiltration.

**Supplementary Figure 3: Diafiltration of recombinant CueO-MacHis with different Molecular Weight Cut Offs (MWCOs).** Vivaspin 30R & 15R: Vivapsin centrifugal concentrators with a Polyethersulfone (PES) membrane and a Molecular Weight Cut Off (MWCO) of 30 or 10 kDa (Sartorius, Göttingen, Germany), M: PagerRuler (Thermo Fisher Scientific), 1: Eluate from an Anion Exchange Chromatography (AEX), 2: Retentate of the Vivaspin diafiltration, 3: Permeate I of the Vivaspin diafiltration.
